# When Strong Recommendations Rest on Weak Evidence: Lessons From Therapeutic Apheresis Guidelines

**DOI:** 10.1002/jca.70098

**Published:** 2026-02-19

**Authors:** Jeremy W. Jacobs, Garrett S. Booth, Brian D. Adkins, Victoria Costa, Sheharyar Raza, Yara A. Park, Joseph Yossi Schwartz, Evan M. Bloch

**Affiliations:** ^1^ Department of Pathology, Microbiology, and Immunology Vanderbilt University Nashville Tennessee USA; ^2^ Department of Pathology University of Texas Southwestern Medical Center Dallas Texas USA; ^3^ Department of Pathology NYU Grossman School of Medicine New York New York USA; ^4^ Department of Laboratory Medicine and Pathobiology University of Toronto Toronto Ontario Canada; ^5^ Canadian Blood Services Medical Affairs and Innovation Toronto Ontario Canada; ^6^ Department of Pathology and Laboratory Medicine University of North Carolina Chapel Hill North Carolina USA; ^7^ Foundation for the Accreditation of Cellular Therapy (FACT) Omaha Nebraska USA; ^8^ Division of Transfusion Medicine, Department of Pathology Johns Hopkins University Baltimore Maryland USA

## Abstract

The American Society for Apheresis (ASFA) guidelines serve as a global standard for therapeutic apheresis practice. However, our analysis of the 2023 guidelines reveals discordance between the strength of recommendation and the quality of evidence. Among 166 indications, one‐third carry strong recommendations, yet only 8% are supported by high‐quality evidence. Over half (55%) are informed by low‐ or very‐low quality evidence. This mismatch is most pronounced for Category I indications, where apheresis is considered first‐line therapy: nearly one‐third are based on low‐quality data, yet 89% receive strong recommendations. Weak evidence is nine times more likely to prompt a strong recommendation for Category I versus Category III indications. This misalignment risks overutilization of apheresis, introduces ethical hurdles for clinical trials by diminishing equipoise, and may mislead patient expectations during informed consent. We advocate for greater transparency by stating the rationale underlying strong recommendations despite low‐quality evidence, acknowledgment of uncertainty where applicable, and suggested research to strengthen the evidence.

Apheresis is a medical procedure where whole blood is removed from an individual and separated into its cellular and plasma components [1]. Specific constituents are extracted through centrifugation or membrane filtration, while the remaining blood is returned to the patient, often with replacement fluids [1]. Therapeutic apheresis is used to treat a variety of conditions including neurological (e.g., myasthenia gravis), hematologic (e.g., thrombotic thrombocytopenic purpura), and renal (e.g., peri‐transplant desensitization and allograft rejection) indications. It is estimated that 250 000–300 000 apheresis procedures are performed annually in the United States alone [2, 3].

The American Society for Apheresis (ASFA) guidelines, which are updated every 3–5 years, have become a global standard for therapeutic apheresis practice. Each clinical indication is assigned both a category that reflects the perceived role of apheresis in management and a GRADE (Grading of Recommendations Assessment, Development and Evaluation) style rating of the strength of recommendation and quality of the available evidence. Indications are categorized from I–IV: Categories I and II represent first‐ and second‐line therapies, respectively; Category III comprises indications for which the optimal role of apheresis has not been established; and Category IV refers to indications where apheresis is considered ineffective or harmful. The two‐tier grading system rates the strength of recommendation (1 being strong and 2 being weak) and the quality of available evidence from A to C corresponding to high, moderate, and low‐ or very‐low quality, respectively [4, 5].

GRADE was developed as a structured, transparent, and reproducible framework for moving from evidence to recommendations, in contrast to earlier consensus‐ or opinion‐based approaches. Within GRADE, strong recommendations are generally expected to rest on high or at least moderate‐certainty evidence, and strong recommendations based on low‐certainty evidence are intended to be rare, “paradigmatic” exceptions that require explicit justification [6, 7, 8]. Accordingly, discordance between a strong recommendation and low‐quality evidence is not simply a matter of opinion, but a defined, analyzable phenomenon within the GRADE framework [9].

A growing literature has systematically examined discordant recommendations across clinical practice guidelines, including those from the Endocrine Society, the World Health Organization, and other specialty societies [10, 11, 12, 13, 14]. These studies have shown that strong recommendations based on low‐quality evidence are common and often inconsistent with GRADE guidance, with important implications for clinical care, patient communication, and research ethics. Building on this work, our goal was to investigate whether similar patterns are present in therapeutic apheresis guidelines and, if so, to characterize their extent and potential consequences.

In this study, we apply pre‐specified, GRADE‐consistent definitions of concordant and discordant recommendations to the 2023 ASFA guidelines. Using the published categories and GRADE ratings, we systematically quantified the alignment between *the strength* of *recommendation* and *quality of evidence* across all 166 indications and examined how this alignment varies by ASFA category. Our intent was not to undermine the clinical judgment of the Writing Committee or to offer purely opinion‐based critique. Rather, we use established methodological criteria from the GRADE framework to identify and describe patterns of discordance that may signal important gaps between the evidence base and the strength of recommendations. We acknowledge that guideline development is not a purely statistical exercise; expert judgment remains indispensable to evidence‐based medicine, which integrates the best available evidence with clinical expertise and patient values [15]. However, appraisal of the available evidence (i.e., GRADE ratings) and the reasoning for the strength of recommendation and Category assignment should be as transparent and reproducible as possible.

One‐third (33.1%, 55/166) of the 166 indications in the 2023 ASFA guidelines have strong recommendations. However, almost a third (30.9%, 17/55) of those strong recommendations are based on low‐ or very low‐quality evidence (Tables 1 and 2). Across all indications, over half (54.8%, 91/166) are informed by low‐ or very‐low‐quality evidence.

**TABLE 1:** Recommendations and evidence quality for 166 indications in the 2023 ASFA guidelines.

	Category I	Category II	Category III	Category IV
Total recommendations (*N* = 166)	27 (16.2%)	44 (26.5%)	91 (54.8%)	4 (2.4%)
Recommendation strength				
Strong (Grade 1) (*n* = 55)	24 (88.9%)	23 (52.3%)	8 (8.8%)	0 (0.0%)
Weak (Grade 2) (*n* = 111)	3 (11.1%)	21 (47.7%)	83 (91.2%)	4 (100.0%)
Evidence quality				
High‐quality (Grade A) (*n* = 14)	5 (18.5%)	4 (9.1%)	5 (5.5%)	0 (0.0%)
Moderate‐quality (Grade B) (*n* = 61)	14 (51.9%)	22 (50.0%)	25 (27.5%)	0 (0.0%)
Low‐ or very‐low quality (Grade C) (*n* = 91)	8 (29.6%)	18 (40.9%)	61 (67.0%)	4 (100.0%)
Recommendation‐evidence grade				
Strong recommendation, high‐quality evidence (Grade 1A) (*n* = 7)	5 (18.5%)	2 (4.5%)	0 (0.0%)	0 (0.0%)
Strong recommendation, moderate‐quality evidence (Grade 1B) (*n* = 31)	13 (48.1%)	15 (34.1%)	3 (3.3%%)	0 (0.0%)
Strong recommendation, low‐ or very‐low quality evidence (Grade 1C) (*n* = 17)	6 (22.2%)	6 (13.4%)	5 (5.5%)	0 (0.0%)
Weak recommendation, high‐quality evidence (Grade 2A) (*n* = 7)	0 (0.0%)	2 (4.5%)	5 (5.5%)	0 (0.0%)
Weak recommendation, moderate‐quality evidence (Grade 2B) (*n* = 30)	1 (3.7%)	7 (15.9%)	22 (24.2%)	0 (0.0%)
Weak recommendation, low‐ or very‐low quality evidence (Grade 2C) (*n* = 74)	2 (7.4%)	12 (27.3%)	56 (61.5%)	4 (100.0%)

**TABLE 2:** Discordant recommendations and strength of evidence in the 2023 ASFA guidelines.

Condition (ASFA fact‐sheet)	Indication (clinical context)	Category	Grade	Standard procedure	Evidence snapshot^a^
Anti‐glomerular basement membrane disease	Diffuse alveolar hemorrhage	I	1C	TPE	~200 pts., 1 small RCT
Wilson disease, fulminant	Bridge to liver transplant	I	1C	TPE	< 100 pts., uncontrolled
Waldenström macroglobulinemia	Prophylactic TPE before rituximab	I	1C	TPE	< 50 pts., no trials
N‐methyl‐D‐aspartate receptor encephalitis	Acute management	I	1C	TPE/IA	> 300 pts., observational
Sickle‐cell disease, acute stroke	Emergency therapy	I	1C	RBC exchange	~240 pts. across 10 series
Transplantation, liver	Desensitization, ABO incompatible, living donor	I	1C	TPE	< 200 pts., no RCTs
Transplantation, heart	Rejection prophylaxis (antibody‐mediated)	II	1C	TPE	> 900 patients in non‐randomized studies
Transplantation, heart	Desensitization	II	1C	TPE	
Transplantation, lung	Chronic lung allograft dysfunction	II	1C	ECP	> 200 patients in > 10 case series
Transplantation, lung	Bronchiolitis obliterans syndrome	II	1C	ECP	
Sickle‐cell disease, acute	Acute chest syndrome (severe)	II	1C	RBC exchange	> 320 patients: 2 non‐randomized trials (*n* = 121) + > 10 case‐series
Neuromyelitis‐optica spectrum disorder	Acute attack/relapse	II	1C	IA	~140 patients: 1 non‐randomized trial (*n* = 61) + 5 case‐series and 17 case‐reports
Chronic acquired demyelinating polyneuropathy	Anti‐myelin‐associated glycoprotein	III	1C	TPE	Small case series
Secondary erythrocytosis	Symptomatic or high‐risk	III	1C	Erythrocytapheresis	~500 cases in 8 series
Hypertriglyceridemic pancreatitis (severe)	Bridge to definitive care	III	1C	TPE/LA	> 300 patients across multiple non‐randomized trials
Progressive multifocal leukoencephalopathy associated with natalizumab	Drug clearance	III	1C	TPE	~300 case reports
Pruritus due to hepatobiliary diseases	Treatment‐resistant	III	1C	TPE	< 30 case reports

^a^ Numbers are taken directly from the evidence tables in each ASFA fact‐sheet and rounded for brevity.

This discordance between recommendation strength and the quality of the supporting evidence is most pronounced for Category I indications, where apheresis is considered as an essential, first‐line therapy. Nearly a third of the 27 Category I indications are based on low‐ or very‐low quality data, yet most (89%) still received a strong recommendation (Tables 1 and 2).

The strength of a given recommendation appears to be influenced by the designated ASFA category, and not solely based on the strength of the evidence alone. For instance, among Category I indications with low‐ or very‐low quality evidence (Grade C), three‐quarters receive strong (Grade 1) recommendations, whereas only 8% of Category III indications that are supported by low‐ or very‐low quality evidence receive a strong recommendation. In short, low‐quality evidence is nine times more likely to be paired with a strong recommendation for a Category I indication than for a Category III indication.

There is also a corollary whereby Category IV indications all have weak recommendations (Grade 2) in the presence of low‐quality evidence (Grade C). Category IV indications are defined as those for which therapeutic apheresis is considered “ineffective or harmful.” In some instances, this designation may be driven, appropriately, by serious or unacceptable adverse events, even when the available efficacy data are sparse and graded as low quality. However, for other indications, the published evidence base pertaining to both the benefits and harm is limited, and the rationale for classifying an intervention as ineffective or harmful is not articulated explicitly. In those cases, clearer documentation of whether the Category IV assignment is based on observed adverse safety signals and/or lack of benefit would help align the “ineffective or harmful” label with the underlying GRADE assessment and increase transparency.

Several factors likely underlie the observed discordance. Methodologically, the ASFA guidelines frequently incorporate case reports and case series, reflecting the reality whereby controlled trials have not been conducted for many rare apheresis indications. In contrast, some prior meta‐analyses of GRADE discordance have centered on guidelines built largely from randomized evidence. For orphan diseases, observational data may therefore represent the entirety of the evidence base. Importantly, this does not, by itself, invalidate strong recommendations. GRADE was developed to support recommendation‐making across evidence levels and permits strong recommendations despite low certainty in specific circumstances, particularly when the condition is serious, the benefits are expected to outweigh the potential harm, and alternatives are limited or inferior [6, 7, 8]. While such exceptions appear uncommon across medical specialties [13], the ASFA context may not be directly comparable because apheresis targets rare, high‐acuity conditions and often lacks feasible trial pathways [14]. Nevertheless, this introduces a self‐perpetuating problem: clinical acuity and the perceived risk of withholding treatment fuel the continued use of apheresis, while dissuading the necessary research to establish its efficacy. Consequently, observational data and/or the biological plausibility of benefit drive many—if not most—apheresis decisions. Indeed, the evidence has not changed for 102 conditions across five successive editions of the ASFA guidelines [16].

The discordance between the strength of recommendation and quality of evidence among Category I indications may reflect a pragmatic approach. In rare, life‐threatening conditions, the same considerations that support labeling apheresis as first‐line therapy—accumulated clinical experience, biological plausibility, limited or inferior alternatives, and practical or ethical barriers to randomized trials—may also justify issuing a strong recommendation despite low‐quality evidence. GRADE explicitly allows for strong recommendations based on low‐certainty evidence in such circumstances, recognizing that clinical expertise, patient values, and contextual factors must inform recommendations alongside empirical data [8, 9]. However, transparency becomes particularly important when both ASFA category assignment and recommendation strength rely heavily on expert consensus.

Only a minority of indications are supported by high‐certainty evidence. Clinical trials are logistically and ethically challenging for rare, severe, or rapidly progressive conditions. The intent is not to impose an unattainable standard; rather it is to ensure that the underlying uncertainty and rationale are explicit when strong recommendations are made on the basis of low‐quality evidence. Likewise, for some indications (e.g., TPE for sepsis with multi‐organ failure, adsorptive cytapheresis for Crohn's disease), moderate to high‐quality evidence may coexist with persistent Category III assignments. This may reflect concerns surrounding implementation and/or uncertainty regarding its benefit; providing the rationale for deferring changes in categories would help clinicians to understand how new evidence is being weighed.

Transparency in the development of guidelines is essential, as recommendations shape both clinical care and research priorities [17]. An explicit rationale for indications with discordance between the strength of the recommendation and the quality of evidence would serve multiple purposes. First, it would help clinicians communicate uncertainty to patients during informed consent. For example, when discussing red cell exchange for acute chest syndrome in sickle cell disease (currently graded 1C), clinicians could explain whether the strong recommendation reflects extensive clinical experience, the life‐threatening nature of untreated disease, the absence of superior alternatives, or a combination of these factors. Second, it would identify areas where the field would benefit from higher‐quality research, potentially facilitating collaborative registry studies or pragmatic trials. Third, it would distinguish between strong recommendations supported by robust observational data versus those based primarily on pathophysiologic reasoning or accumulated clinical experience.

Future ASFA guidelines could include a supplementary table (or appendix) documenting the primary rationale for each Category I and II indication, particularly those with Grade 1C assignments. In parallel, registries, pragmatic studies, and platform trials could strengthen the evidence base, and incorporating international practice patterns and health‐system capacity may improve generalizability of future guideline iterations [18, 19]. Acknowledging uncertainty explicitly offers dual benefit: it would facilitate clinical decision‐making through greater awareness of a nuanced risk–benefit calculus while encouraging investigation, collectively forging a stronger foundation for this important therapeutic modality.

## Funding

E.M.B.'s effort is supported in part by the National Heart, Lung, and Blood Institute (1K23HL151826).

## Ethics Statement

The authors have nothing to report.

## Conflicts of Interest

E.M.B. reports personal fees and non‐financial support from Terumo BCT, Grifols, Abbott Laboratories, and UptoDate, outside of the submitted work. E.M.B. is a member of the United States Food and Drug Administration (FDA) Blood Products Advisory Committee. Any views or opinions that are expressed in this manuscript are those of the author's, based on his own scientific expertise and professional judgment; they do not necessarily represent the views of either the Blood Products Advisory Committee or the formal position of the FDA, and do not bind or otherwise obligate or commit either Advisory Committee or the Agency to the views expressed.

## Data Availability

All data from which the analyses presented in this study were derived are publicly available in the ASFA Guidelines.
